# Therapeutic Strategies and Chemoprevention of Atherosclerosis: What Do We Know and Where Do We Go?

**DOI:** 10.3390/pharmaceutics14040722

**Published:** 2022-03-28

**Authors:** Ana Clara Aprotosoaie, Alexandru-Dan Costache, Irina-Iuliana Costache

**Affiliations:** 1 Faculty of Pharmacy, Grigore T. Popa University of Medicine and Pharmacy Iasi, 700115 Iasi, Romania; ana.aprotosoaie@umfiasi.ro; 2 Department of Cardiovascular Rehabilitation, Clinical Rehabilitation Hospital, 700661 Iasi, Romania; 3 Department of Internal Medicine I, Faculty of Medicine, University of Medicine and Pharmacy “Grigore T. Popa”, 700115 Iasi, Romania; irina.costache@umfiasi.ro; 4 Department of Cardiology, “St. Spiridon” Emergency County Hospital, 700111 Iasi, Romania

**Keywords:** atherosclerosis, pharmacotherapy, emergent therapeutics, natural anti-atherosclerotic products

## Abstract

Despite progress in understanding the pathogenesis of atherosclerosis, the development of effective therapeutic strategies is a challenging task that requires more research to attain its full potential. This review discusses current pharmacotherapy in atherosclerosis and explores the potential of some important emerging therapies (antibody-based therapeutics, cytokine-targeting therapy, antisense oligonucleotides, photodynamic therapy and theranostics) in terms of clinical translation. A chemopreventive approach based on modern research of plant-derived products is also presented. Future perspectives on preventive and therapeutic management of atherosclerosis and the design of tailored treatments are outlined.

## 1. Introduction

Atherosclerosis is currently recognized as a progressive metabolic and immune-inflammatory disease that affects the intimal lining of medium- and large-sized arteries [1,2,3]. It is considered to be the main cause of atherosclerotic cardiovascular diseases—a category that include coronary artery disease, cerebrovascular disease and peripheral artery disease [4]. Ischemic heart diseases and stroke are the major atherosclerotic cardiovascular events, representing 85% of cardiovascular deaths and 28% of all-cause mortality [5]. According to the Global Burden of Cardiovascular Diseases and Risk Factors Study, 1990–2019, cardiovascular diseases affected 523 million patients globally and were the underlying cause of 18.6 million deaths—approximately one-third of all deaths worldwide [6,7]. Although significant advances have been made in the diagnosis and treatment of atherosclerosis and its pathological mechanisms, subsequent complications and importance in the field of medicine, many aspects are yet to be known and elucidated; in particular, pharmacotherapy of atherosclerosis is a challenging task. The pathogenesis of atherosclerosis is complicated and multifactorial, involving interplay between the cellular structure of the arterial intima, lipid metabolism and inflammatory factors [8,9,10]. After historically being localized mainly in the Western countries, atherosclerosis has spread worldwide and to younger people, females and all races. Further, in addition to the traditional risk factors (age, males older than 45 years and females older than 55 years, family history of premature cardiovascular diseases, arterial hypertension, hypercholesterolemia, diabetes, smoking and obesity/overweight), new risks factors have been identified, such as genetics, homocysteine, high-sensitivity C-reactive protein (hs-CRP), sleep disorders, sedentary lifestyle, air pollution and environmental stress [4,9,11]. Despite considerable progress in atherosclerosis research, current therapies are insufficient to effectively treat this disease. It is appreciated that the currently available medicines cannot prevent the occurrence of even 70% of clinical events [12]. There is a need for effective preventive strategies and better therapeutic options for atherosclerosis. This review discusses mechanisms, clinical efficacy and limitations and current evidence for atherosclerosis therapy starting from classical pharmacotherapy (statins, fibrates and cholesterol absorption inhibitors) to emerging therapies (antibody-based therapeutics, cytokine-targeting therapy, antisense oligonucleotides, photodynamic therapy and theranostics). Further, alternative chemopreventive strategies based on natural products are explored. Future perspectives on the preventive and therapeutic management of atherosclerosis and the design of tailored treatments are outlined.

## 2. Pathogenesis of Atherosclerosis

**Figure 1:**
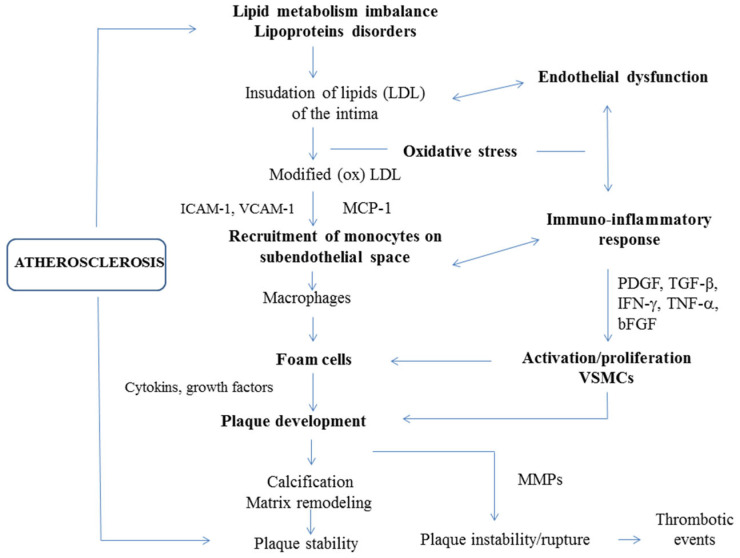
The pathogenesis of atherosclerosis. Atherogenesis is linked to both lipid metabolism and immune-inflammatory pathways. The process which leads to the formation of atherosclerotic plaques is complex and involves multiple steps. Endothelial dysfunction—induced by various noxious stimuli, including cardiovascular risk factors, disturbed hemodynamics and/or high levels of circulating lipids, particularly LDL—is a key component in the onset of atherosclerosis. Subendothelial accumulation of LDL and its oxidative modification to oxLDL promotes endothelial injury and induces a complex picture of immuno-inflammatory responses, starting from monocyte recruitment and macrophage activation, which lead to foam-cell formation and atherosclerotic plaque development. Monocyte chemoattractant protein 1 (MCP-1) and adhesion molecules (vascular cell adhesion molecule-1 (VCAM-1) and intercellular adhesion molecule-1 (ICAM-1)) are mainly responsible for monocyte chemotaxis and adhesion. Atherogenic activation and phenotypic switching of vascular smooth muscle cells (VSMCs) induced by pro-inflammatory signaling (interferon γ (IFN-γ) and tumor necrosis factor α (TNF-α)) and growth factors (platelet-derived growth factor (PDGF), transforming growth factor β (TGF-β) and basic fibroblastic growth factor (bFGF)) also contribute to foam cell and plaque formation. Calcification and extracellular matrix remodeling may stabilize atherosclerotic plaques. Macrophages and foam cells release matrix metalloproteinases (MMPs) that promote the rupture of plaques and lead to major thrombotic events [13,14].

Pathobiology of atherosclerosis involves crosstalk between lipid metabolism imbalance, endothelial dysfunction, inflammatory pathways, oxidative stress and genetic predisposition. Endothelial cells, inflammatory cells and vascular smooth muscle cells (VSMCs) play key roles in disease development and progression. The main events that occur in atherogenesis are illustrated in Figure 1.

### 2.1. Endothelial Cells

Healthy endothelium is a major regulator of vascular homeostasis, releasing a wide spectrum of factors that control vascular tone and permeability, fluid balance, cellular adhesion, thromboresistance and fibro-inflammatory-proliferative responses. Endothelial-derived nitric oxide (NO) is a major factor that promotes vasoprotective and anti-atherosclerotic behavior [15,16]. Classical (hypercholesterolemia, hypertension, diabetes, chronic smoking and aging) and non-classical (ambient air pollution, mental stress and chronic inflammatory diseases) cardiovascular risk factors alter endothelial function, switching from a quiescent phenotype toward one that involves the impairment of NO-dependent signaling and pro-inflammatory and pro-thrombotic maladaptive responses [17]. Damage to endothelial function is an early hallmark of atherosclerosis, but it also has diagnostic and therapeutic significance [17]. Dysfunctional endothelial cells (ECs) exhibit increased permeability, a vasoconstrictive profile and express leukocyte adhesion by releasing adhesion molecules (vascular cell adhesion molecule-1 (VCAM-1), intercellular adhesion molecule-1 (ICAM-1) and E- and P-selectin); also, the pro-inflammatory nuclear factor-κB (NF-κB) and activator protein 1 (AP-1) pathways are activated, and the cytoskeletal and junctional proteins are dysregulated [13,18]. In addition, ECs showed an altered profile of gene expression and repair [19]. This defective endothelial phenotype allows subendothelial accumulation of low-density lipoproteins (LDL) and their atherogenic modification by oxidation and aggregation [20]. Locally reactive oxygen species (ROS) and myeloperoxidase, 15-lipoxigenase and nicotinamide adenine dinucleotide phosphate (NADH/NADPH) oxidases appear to mediate oxidation of LDL [21]. Oxidized LDL (oxLDL) contributes to the development and progression of atherosclerosis through induction of inflammatory and immune cell infiltration into the vascular wall, increase of oxidative stress and upregulation of the renin–angiotensin system [21].

### 2.2. Inflammatory Cells

*Monocytes/macrophages.* Recruitment of circulating monocytes within the intima is elicited by the retention of lipoproteins and is one of the earliest atherogenic events; this process continues in subsequent stages of disease progression. Monocyte chemoattractant protein 1 and 3 (MCP-1 and -3) chemokines are implicated in monocytic chemotaxis to damaged vascular areas [22]. In the subendothelial space, monocytes differentiate into macrophages, which engulf the modified LDL via several scavenger receptors (SR-A1, CD36 and LOX1/SR-E1) and lead to the formation of foam cells [23]. Apart from their role as scavengers, the activated macrophages contribute to the release of vasoactive factors (endothelins, eicosanoids, ROS, chemokines, tissue factor (TF) and matrix metalloproteinases (MMP-2, MMP-8, MMP-12)) [14,20,24] that exhibit thrombogenic, inflammatory and plaque-destabilizing properties and accelerate the progression of atherosclerotic lesions [20,25]. Due to their high plasticity, macrophages can adopt different functional phenotypes as a result of their adaptation to the local environment, such as intracellular energy metabolism [26,27]. Genetic and epigenetic variables—including non-coding RNA and gut microbiota—can also influence macrophage function, influencing both inflammatory response and resolution/repair [27]. The traditional model of macrophage polarization includes two phenotypes: M1 and M2. “Classic” M1 macrophages are triggered by Th1 cytokines (interferon-γ (IFN-γ) and tumor necrosis factor α (TNF-α)) and molecular complexes associated with pathogens (lipopolysaccharides). They exhibit an inflammatory behavior, increasing levels of IL-6, IL-12, IL-23, TNF-α and IL-1β cytokines and chemokines involved in Th1 recruitment (CXCL-9, CXCL-10 and CXCL-11). In addition, M1 macrophages produce RSO via NADPH oxidase and stimulate tissue destruction [26]. “Alternative” M2 macrophages are usually polarized by Th2-related cytokines such as IL-4, IL-33 and IL-13. They express immunomodulatory properties, stimulate tissue recovery and secrete anti-inflammatory cytokines (IL-10) and chemokines (CCL17, CCL22, CCL24) [26]. The M1 type is commonly encountered in atherosclerotic areas in patients with coronary heart disease and heart attack, being prevalent in vulnerable plaques [26]. Depending on the stimuli, other macrophage phenotypes have been described in atherosclerosis. Mox macrophages are triggered by oxidized phospholipids and are characterized as a proatherogenic type that is abundant in advanced plaques [23]. Further, the so-called M4 phenotype shows a pro-inflammatory and proatherogenic profile, being polarized by the CXCL4 chemokine. It promotes destabilization of the plaque’s fibrous cap and appears to be irreversible in atherosclerotic plaques. Mhem and M(Hb) are bleeding-related phenotypes that exhibit atheroprotective properties, promoting cholesterol outflow and resistance to the formation of foam cells [23,26,28].

*Lymphocytes.* Immunoregulatory CD4+ and CD8+ T cells are encountered in all stages of atherosclerotic plaque development and progression. On the other hand, B lymphocytes are rare in the intimal plaque [20]. The differentiation of T cells into functional types depends on local metabolic and systemic conditions, such as hypoxia, cellular energy metabolism, cellular cholesterol efflux, hypercholesterolemia and epigenetic changes [27].

CD4+ T-helper (Th) cells represent about 70% of T lymphocytes in atherosclerotic areas, with the Th1 subtype predominant. Th1 cells secrete IFN-γ, TNF-α and IL-2 cytokines, which enhance the atherogenic processes [22,29]. CD8+ T cells are commonly found in severe advanced lesions and appear to exhibit proatherogenic and plaque-destabilizing properties [30]. The Th2 subtype shows both proatherogenic and protective effects. Thus, its related IL-4 cytokines contribute to plaque progression, while IL-5 and IL-33 exhibit anti-atherogenic properties via the production of IgM-type anti-oxLDL antibodies and reduction of the size of the atherosclerotic area. Induction of Th2 cells is pronounced in severe hypercholesterolemia [29].

CD4+ regulatory T cells (Tregs) represent 1–5% of all localized T cells within the atherosclerotic plaque. They exert immunosuppressive effects and are master modulators of inflammatory responses. A prevalent atheroprotective function has been documented for Tregs-related cytokines (IL-10 and transforming growth factor (TGF-β)) [29,31].

The T helper 17 (Th17) cells have been also been identified in atherosclerotic zones. They produce IL-17A that promotes autoimmunity but also induces modification of atherosclerotic plaque. On the one hand, IL-17A can enhance recruitment and activation of myeloid cells, while on the other hand, it stabilizes plaque via stimulation of collagen production. Therefore, its role is controversial [32].

*Dendritic cells.* Dendritic cells exert both direct and indirect effects in atherogenesis. Direct actions include lipid uptake and transformation in foam cells, antigen presentation and activation/proliferation of T cells and mediation of efferocytosis, a phagocytic clearance of apoptotic cells. Indirect effects are related to regulation of other immune cell functions and recruitment of circulating leucocytes or hematopoietic cells to the vascular area via secretion of pro-inflammatory mediators (TNF-α, IL-1, IL-12, IL-23, IL-27 and IL-33). [33].

### 2.3. Vascular Smooth Muscle Cells (VSMCs)

VSMCs are involved in the development of atherogenesis through their migration and proliferation and induction of matrix synthesis and foam cell formation. Even VSMC apoptosis and senescence contribute to atherosclerosis [24]. VSMCs are activated in response to various factors (TNF-α, IFN-γ, TGF-β, platelet derived growth factor (PDGF) and basic fibroblastic growth factor (bFGF)), ROS, shear stress, blood flow, matrix stiffness and modified cholesterol); once activated, they switch from a contractile phenotype to a synthetic state that promote extracellular matrix (ECM) production, plaque growth and fibrotic cap formation [18]. The vulnerable thinning of the fibrous cap as a result of defective efferocytosis and cell necrosis can cause rupture of the plaque and thrombosis, which further triggers severe cardiovascular events (unstable angina, myocardial infarction, stroke and sudden cardiac death) [34]. VSMCs genes associated with differentiation, migration and phenotypic switching are transcriptionally regulated by epigenetic mechanisms such as DNA methylation [24]. 

The role of epigenetics in regulating the atherosclerotic process has been increasingly recognized, with DNA methylation most commonly associated, as methylation levels control the expression of pro- and anti-atherosclerosis genes [35]. Further, non-coding RNA participates in the regulation of apoptosis, pyroptosis, autophagy, proliferation and migration of endothelial cells, monocytes, macrophages and VSMCs, making it a component of the atherosclerotic cycle and a target for future therapy [36].

### 2.4. Risk Factors

As we mentioned before, atherogenic changes are sustained and magnified by the influence of various classical (conventional) and non-classical (non-traditional) risk factors. A brief description of atherogenic mechanisms induced by the major factors is presented below.

#### 2.4.1. Classical Risk Factors

##### Hypercholesterolemia

High levels of total cholesterol and triglycerides (TG) plus lipoprotein imbalance (with the prevalence of atherogenic fractions (LDL) and decrease of high-density lipoproteins (HDL)) play a crucial role in atherosclerosis, initiating the pathological processes we previously described. They are associated with the impairment of endothelium-dependent vasodilatation and ECs function and repair, abnormal vasomotor response, disturbed hemodynamics, oxidative stress and inflammation [37,38]. Familial hypercholesterolemia is an inherited hyper-LDL-cholesterolemia caused by deleterious mutations in LDL receptors or genes [39]. Hepatic LDL receptors are important for the removal of plasma cholesterol. The binding of LDL to its receptors is mediated by apolipoprotein B, while proprotein convertase subtilisin-kexin type 9 (PCSK9) is responsible for the degradation of LDL receptors. Mutations of genes that code LDL receptors, apoliprotein B or PCSK9 may result in minimal cholesterol clearance, leading to extensive accumulation [40]. 

The control of circulating cholesterol is one of the most-used targets of current anti-atherosclerotic pharmacotherapy and dietary recommendations.

*Hypertension.* Elevated blood pressure accentuates the progression of atherosclerosis, increasing its severity and expanding lesions. The deleterious effects of arterial hypertension are due to multiple mechanisms, including endothelial dysfunction induced by mechanical and hemodynamic stress, increase of atherogenic lipoprotein uptake into the intima and monocyte adherence to the endothelial line. Further, vascular inflammation, promotion of the synthetic state of VSMCs and the activation of prothrombotic factors via angiotensin (Ang) II and other vasoconstrictive peptides play significant roles [41,42,43]. In addition to its established role in hypertension, the renin–angiotensin system (RAS) has major implications in atherosclerosis pathogenesis via various cellular and molecular mechanisms. Ang II, the main effector of RAS, induces oxidative stress in the cardiovascular system, significantly alters ECs function, upregulates the expression of adhesion molecules (ICAM-1, VCAM-1) and inflammatory cytokines (TNF-α, IL-6) and stimulates growth factor expression (insulin-like growth factor and platelet-derived growth factors). Ang II stimulates plaque formation in the early stages and promotes plaque progression; in advanced disease, it destabilization the atherosclerotic plaque by regulating ECM composition and MMP release [44].

*Diabetes*. Diabetes mellitus increases the incidence of atherosclerosis and accelerates the progression and the clinical manifestation of atherosclerotic pathology. More than 90% of patients with diabetes and atherosclerosis are diagnosed with type 2 diabetes, which is generally known to amplify the risk of cardiovascular complications and mortality. Diabetes-related metabolic abnormalities, such as chronic hyperglycemia, advanced glycation end-products, dyslipidemia, free fatty acid excess and insulin resistance, cause a cascade of modifications that impact multiple cell types and alter vascular homeostasis [45]. They impair endothelial cell function, promote vasoconstriction and thrombosis, increase proinflammatory signaling, enhance foam cell formation and stimulate atherogenic VSMCs behavior [45,46]. Prediabetic patients are also at risk. Hyperglycemia leads to increased hematopoiesis and ROS-producing neutrophils, as well as to the production of extracellular vesicles from vascular endothelial cells and leukocytes, which, in turn, facilitate atherosclerosis via ROS-producing NADPH oxidase and LDL-scavenging CD36. They also carry specific miRNAs that promote hematopoiesis and inflammation [47].

*Smoking.* Chronic smoking is a major cause of cardiovascular morbidity and mortality. It triggers atherothrombotic events and promotes atherosclerosis and contributes to all of its clinical expressions. Tobacco exposure (even passive smoking) is associated with vasomotor dysfunction and endothelial cell damage, inflammation (elevated levels of TNF-α, IL-6 and CRP), dysfunctional thrombo–hemostatic response via alterations in platelet activity and antithrombotic/prothrombotic factors, impairment of lipid profile and lipid peroxidation and VSMCs proliferation [48,49].

*Aging*. Atherosclerosis is more frequently encountered in the older population, being linked to changes in both myeloid cell hematopoiesis and vasculature, as these systems share the same inflammatory pathway mediated by IL-6 signaling. This highlights the role of anti-inflammatory therapies in the prevention of atherosclerosis [50]. Aging is an independent risk factor for atherosclerosis and adverse cardiovascular outcomes. All atherogenic events are amplified with increasing age. The most important age-related changes that support and promote atherosclerosis include vascular stiffness and rigidity, endothelial cell injury, persistent vascular inflammation and oxidative stress, increased of atherogenic lipoprotein uptake and leukocyte adhesion, hypertension, alteration of mitochondrial function in vasculature and mitophagy impairment. Elevated DNA damage, extensive telomere shortening, epigenetic alteration and gene transcription dysregulation are characteristic features of both aged cells and atherosclerotic plaques [50,51].

#### 2.4.2. Non-Classical Risk Factors

*Hyperhomocysteinemia*, defined as homocysteine values higher than 10^−2^ mol/L, is a new, independently important risk factor for atherosclerosis and cardiovascular disease [52]. Elevated levels of plasma homocysteine may arise from abnormalities of enzymes involved in its metabolism or nutritional deficiencies of folate, B6 and/or B12 vitamins. Older age, tobacco use, hypertension and male gender are associated with increased homocysteine levels. Further, patients with renal dysfunction, malignant neoplasm and systemic lupus erythematosus show the same tendency [53]. Hyperhomocysteinemia promotes a proatherogenic and prothrombotic microenvironment via several mechanisms that include oxidative stress, endoplasmic reticulum stress, endothelial dysfunction, increase of platelet aggregation, alteration of fibrinolysis, activation of proinflammatory cytokine production and stimulation of VSMCs proliferation [52,53]. It is important to mention that hyperhomocysteinemia reinforces the effects of other risk factors, such as smoking, hypertension and lipid metabolism imbalance [54].

*Bacterial and viral infections*.

Infection with a variety of pathogens (*Chlamydia pneumoniae*, periodontal pathogens, *Helicobacter pylori*, Human Immunodeficiency Virus, Influenza virus and Cytomegalovirus) may contribute to the development of atherosclerosis triggering and enhancing the systemic inflammatory response. Elevated CRP levels as a marker for predictive atherosclerotic risk have been reported in these patients. Besides, other mechanisms that promote atherogenesis can be also involved, such as: endothelial damage, altered cell metabolism, monocyte and macrophage activation, dysregulated lipid metabolism and hyperhomocysteinemia [48]. Severe Acute Respiratory Syndrome Coronavirus-2 (SARS-CoV-2), responsible for the 2019 coronavirus pandemic (COVID-19) is also strongly associated with atherosclerotic cardiovascular diseases and their complications. The suggested mechanisms include cytokine storm (TNF-α, IL-1β, IL-2, IL-6 and CRP) and thrombotic microangiopathy [55].

*Environmental Pollutants*.

Epidemiological studies show a consistent relationship between atmospheric pollutants (particulate matter and gaseous products) exposure and atherosclerotic cardiovascular diseases. Air pollutants generated by anthropogenic activities (fossil fuels, refining and construction) and natural sources (volcanic eruptions and wildfires) can induce atherogenic responses via several mechanisms, such as: endothelial dysfunction, alteration of systemic micro- and macrovascular tone, hyperlipidemia, tissue inflammation, increase of thrombogenicity, oxidative and neuroendocrine stress, tissue damage and plaque instability. It is important to mention that air pollution exposure amplifies the effects of other cardiovascular risk factors (hypertension, insulin resistance and aging) [48,56].

Some autoimmune disorders (psoriasis, rheumatoid arthritis, systemic lupus erythematous and antiphospholipid syndrome), mental health disorders (chronic depression), sleep disorders and polycystic ovarian syndrome have also been linked to an increased atherosclerotic and cardiovascular risk. Although the mechanisms are not fully understood, chronic inflammation, oxidative stress and neuro-endocrine abnormalities may contribute significantly [48].

Given the higher prevalence of atherosclerosis in men as compared to women in the pre-menopause age, plus the sudden rise of incidence in women after menopause, together with the abundance of sex hormone receptors in the vascular endothelium, sex hormones also play a role in atherosclerosis development, and the onset of menopause represents another risk factor [57].

### 2.5. Groups Prone to Atherosclerosis

Several population groups are at a higher risk of developing atherosclerosis or a disease associated with it. These include: familial dyslipidemia, women, older people, type 2 diabetes mellitus, metabolic syndrome, patients with acute coronary syndromes, stroke, chronic kidney disease, transplantation, peripheral artery disease, chronic immune-mediated inflammatory diseases, patients with Human Immunodeficiency Virus (HIV) and patients with severe mental illness. Familial dyslipidemias are associated with genetic syndromes, the most frequent and studied being familial hypercholesterolemia. Usually, they imply the alteration of lipid metabolism and a similar lipid profile in many members of the same family. Women have a lower risk than men before the onset of menopause; however, after the age of 55, the risk is equal in both genders, and cardiovascular mortality tends to be higher in women. The use of statins has not been fully studied in patients >75 years old and thus there is no full recommendation for treatment to be initiated. Further, given the lower treatment adherence in this group and high likelihood of other comorbidities, cardiovascular risk is also higher. Type 2 diabetes mellitus is associated with organ damage (nephropathy and retinopathy neuropathy), dyslipidemia, obesity and hypertension, which significantly increase the risk for atherosclerotic cardiovascular disease. Patients who have already suffered major events such as coronary syndromes or strokes (which also have a major atherosclerotic etiology) are at a higher risk of reoccurrence plus have more difficulty reaching and maintaining LDL target values. Chronic kidney disease is itself a major cardiovascular risk factor, and it is associated with increased atherosclerosis. From the early stages, TG levels rise and HDL levels are low, while small, dense LDL particles become predominant with disease progression. Post-transplant patients (especially heart, lung, liver, kidney or allogenic hematopoietic stem cell transplantation) as well as those who have undergone immunosuppressive therapy have an altered lipid profile, with increased total cholesterol, very-low-density lipoprotein (VLDL) and TG levels. Further, interactions between immunosuppressive medication and lipid-lowering drugs should be taken into consideration. Peripheral artery disease is already a manifestation of atherosclerosis, yet it is always associated with an increased risk for major events, such as strokes or coronary atherosclerotic syndromes [58].

## 3. Current Pharmacotherapy in Atherosclerosis

Currently available anti-atherosclerotic pharmacotherapy includes mainly lipid-lowering agents, namely statins, fibrates, cholesterol-absorption inhibitors and proprotein convertase subtilisin/kexin type 9 (PCSK-9) inhibitors [59]. Antiplatelet and antihypertensive drugs are also prescribed. 

### 3.1. Statins

Statins are the most-commonly prescribed drugs, being first-line therapy for atherosclerosis and clinical management of the cardiovascular risk. They are effective both in primary and secondary prevention of cardiovascular disease [60]. The atheroprotective activity of statins involves both potent LDLc-lowering properties and multiple non-lipid-related pleiotropic effects, including enhancement of nitric oxide (NO) bioavailability, alleviation of endothelial dysfunction, anti-inflammatory, immunomodulatory and antioxidant abilities, stabilization of atherosclerotic plaques and inhibition of cardiac hypertrophy [61]. The major mechanism of statins is competitive and reversible inhibition of 3-hydroxy-3-methyl glutaryl coenzyme A (HMG-CoA) reductase, a rate-limiting enzyme in the cholesterol biosynthesis pathway. The decrease of cholesterol levels leads to upregulation of LDL-receptor expression and increased bloodstream LDLc clearance, reducing circulating LDLc by 20–55% [60]. Further, statins inhibit hepatic synthesis of apolipoprotein B100 and decrease production of triglyceride-rich lipoprotein [62]. They can alter plaque biology and reduce foam cell formation via suppression of oxLDL uptake by CD36, scavenger receptor A and LOX-1 receptor and inhibition of macrophage oxidative activity [62,63]. Other beneficial effects of statins include inhibition of endothelial nitric oxide synthase (eNOS), decrease of CRP levels, reduction of adhesion molecules (E-selectin and ICAM-1), suppression of myeloperoxidase-derived and nitric oxide-derived oxidants, upregulation of key antioxidant enzymes (glutathione peroxidase and superoxide dismutase) and recruitment of endothelial progenitor cells (useful in repairing ischemic injuries) [61,64]. Clinical statins are simvastatin, lovastatin, pravastatin, fluvastatin, atorvastatin, rosuvastin and pitavastatin [62]. The most important side effects of statin therapy are muscle symptoms (myalgia and rhabdomyolysis), liver dysfunction and renal failure. [60,65].

### 3.2. Fibrates

These lipid-lowering agents attenuate premature atherosclerosis and cardiovascular risk in atherogenic dyslipidemias, including those from type II diabetes and metabolic syndrome [66]. Fibrates are considered an effective therapeutic strategy in patients with moderate to high residual cardiovascular risk, mainly those with hypertriglyceridemia and low high-density lipoprotein cholesterol (HDLc) values [67]. They decrease plasma triglycerides (TG) and TG-rich lipoproteins and increase HDLc, primarily through the activation of peroxisome proliferator-activated receptor α (PPARα), a master transcription factor that regulates lipid and carbohydrate metabolism, impacting fatty acid uptake and activation, TG turnover, lipid droplet biology and gluconeogenesis [68,69]. Additional anti-atherogenic activity of fibrates includes anti-inflammatory effects and the decrease of vascular cell adhesion molecule (VCAM) and MCP-1 levels [67]. The best-known fibrates are fenofibrate, gemfibrozil and bezafibrate. Common adverse effects associated with fibrates are gastro-intestinal, liver and musculoskeletal disturbances [59].

### 3.3. Cholesterol Absorption Inhibitors

Ezetimibe is the commonly used drug of this type. It inhibits intestinal absorption of dietary and biliary cholesterol and related phytosterols, preventing transport of cholesterol through the intestinal wall [70]. Its association with simvastatin enhances the lipid-lowering effect of ezetimibe. The molecular target of the drug is Niemann–Pick C1-Like 1 protein, a critical cholesterol-trafficking factor [59,71]. 

### 3.4. Proprotein Convertase Subtilisin/Kexin Type 9 (PCSK-9) Inhibitors

PCSK-9 is another relevant cholesterol-lowering target that is involved in the regulation of LDL receptors. The gene that encodes PCSK-9 is implicated in familial hypercholesterolemia, and their gain- and loss-of-function mutations lead to high and low levels of LDLc, respectively. Alirocumab and evolocumab are two human monoclonal antibodies that act as PCSK-9 inhibitors. They are approved in the treatment of familial hypercholesterolemia and in patients with atherosclerotic cardiovascular disease who require additional LDLc reduction. 

Further, inclisiran is a small interfering RNA molecule (siRNAs) that suppresses PCSK-9 synthesis in hepatocytes. It specifically binds to PCSK-9 mRNA, inhibits its translation and switches off PCSK-9 synthesis, resulting in a substantial and long-lasting decrease of serum LDLc levels. Analysis of data from three clinical trials showed that inclisiran decreased LDLc concentration by 51%, total cholesterol by 37%, ApoB by 41% and lowered the incidence of significant negative cardiovascular events by 24% [12]. The main therapeutic indications of inclisiran are primary hypercholesterolemia and mixed dyslipidemia in monotherapy or combined with statins or other lipid-lowering agents in patients who cannot achieve LDLc goals. Although the drug has an acceptable side effects–benefits profile, it should be noted that the potential pro-thrombotic activity of inclisiran can become clinically relevant after a long period of use in patients with high cardiovascular risk [72].

### 3.5. Renin–Angiotensin System (RAS) Inhibitors

RAS can be inhibited using three main types of agents: direct renin inhibitors (e.g., Aliskiren), angiotensin converting enzyme (ACE) inhibitors (captopril, enalapril and perindopril) and angiotensin receptor blockers (ARBs) (losartan, candesartan and irbesartan). Apart from their main activity, these drug classes can also have a hypolipemic effect, as atherosclerosis is linked to the renin–angiotensin system, as previously mentioned. Prevention of post-vascular injury myointimal proliferation, inhibition of Ang II-induced vascular proliferation or simply the lowering of blood pressure can decrease atherosclerosis formation. Further, compared to other blood-pressure-lowering classes (beta-blockers and calcium channel blockers), only RAS inhibitors exhibit anti-atherosclerotic properties. However, it should be noted that these drug classes are foremost blood-pressure-lowering medication, and the lipid-lowering properties are only potential additional effects [73,74,75,76,77].

## 4. Emerging Therapies

### 4.1. Cytokine-Targeting Therapy 

Since atherosclerosis is no longer considered just a lipid-derived disease, and the role of inflammation in the atherosclerotic process has been revealed, strategies targeting inflammatory pathways were developed as promising new avenues to treat this pathology. The most-investigated inflammatory targets have been IL-1β, IL-6, CRP, TNF-α and IFN-γ, and the promising therapeutics are presented below [78].

#### 4.1.1. Anti-IL-1β Agents

Cytokine IL-1β is involved in all stages of atherosclerosis, being a major pathogenic factor in plaque instability. It increases expression of chemokines (MCP-1) and adhesion molecules (ICAM-1, VCAM-1), stimulates proliferation and differentiation of vascular smooth muscle cells, and induces the activation of macrophages and the secretion of different proinflammatory mediators (IL-6) and MMPs [79]. Canakinumab is a recombinant human monoclonal antibody that was approved for the treatment of cryopyrin-associated periodic syndrome, adult-onset Still’s disease, systemic juvenile idiopathic arthritis or familial Mediterranean fever. It binds to IL-1β and blocks its interaction with the IL-1 receptor, neutralizing IL-1β signaling [80]. In the CANTOS study (Canakinumab Anti-inflammatory Thrombosis Outcome Study), involving patients with a history of myocardial infarction and a high-sensitivity CRP level (≥2 mg/L), canakinumab therapy led to a 15% reduction in major cardiovascular events (nonfatal myocardial infarction, nonfatal stroke and cardiovascular death). Furthermore, in a pre-specified secondary analysis of trial results, all-cause and cardiovascular mortality decreased by 31% in treated patients, regardless of the reduction in lipid levels. The safety profile of canakinumab is favorable, but it can increase the risk of infections and could promote plaque instability [79,81]. Another known IL-1β blocker is anakinra, a recombinant IL-1 receptor antagonist used in therapy of rheumatoid arthritis and neonatal-onset multisystem inflammatory disease [80]. In clinical trials in patients with acute coronary syndrome, anakinra significantly reduced the acute inflammatory response and subsequent cardiovascular events and hospitalizations. Due to side effects related to frequent administration, however, the drug is not appropriate for chronic disease management [79].

#### 4.1.2. Anti-IL-6 Agents

IL-6 is a pleiotropic and potent cytokine, being a marker of inflammation related to cardiovascular risk. Elevated IL-6 levels are associated with increased cardiovascular events and mortality. Pro-atherogenic effects of IL-6 include increase of amyloid protein, fibrinogen, adhesion molecules (ICAM-1, VCAM) and CRP expression, activation of endothelial cells and platelets, stimulation of vascular smooth muscle proliferation and macrophage lipid accumulation. In addition, IL-6 promotes progression of coronary artery disease [82,83]. There are also data that support some atheroprotective properties of IL-6 via upregulation of ATP binding cassette transporter (ABC)A1, a protein that mediates cholesterol efflux from cells to apolipoproteins and prevents foam cell formation. Tocilizumab is a recombinant humanized monoclonal antibody that interferes with the binding of IL-6 to its specific receptors on different cell types. It prevents inflammatory responses and is recommended in moderate to severe rheumatoid arthritis, systemic juvenile idiopathic arthritis and in COVID patients. Clinical studies have shown some cardiovascular benefits of tocilizumab. In patients with non-ST-segment elevation myocardial infarction (NSTEMI), tocilizumab reduced CRP levels by up to 50%. Further, it decreased troponin T values in patients who underwent percutaneous coronary intervention (PCI) and improved myocardial salvage after STEMI (ASSAIL-MI trial) [84]. Unfortunately, tocilizumab causes adverse changes in the lipid profile, raising LDL and TG via reductions in LDL receptor levels. Currently, ziltivekimab, a novel human monoclonal antibody targeting the IL-6 ligand, is being developed for atherosclerosis treatment. Phase 2 of the RESCUE trial reported that subcutaneous administration of ziltivekimab in patients with high cardiovascular risk significantly reduced biomarkers of systemic inflammation and thrombosis known to promote the atherothrombotic process (hsCRP, fibrinogen, serum amyloid A, secretory phospholipase A2 and LP(a)). Treatment was well-tolerated and had no influence on the total cholesterol to HDLc ratio [12,85].

#### 4.1.3. Anti-TNF-α Agents

TNF-α is a master regulator of inflammatory events and immune responses and a major player in atherogenesis. It induces endothelial barrier dysfunction by increasing vascular permeability, decreases NO bioavailability, stimulates vascular ROS generation (primarily anion superoxide), promotes endothelial expression of cell adhesion molecules (E-selectin, VCAM-1 and ICAM-1) and stimulates the recruitment and migration of leukocytes in the vascular wall. Moreover, TNF-α alters smooth muscle cell function by stimulating their proliferation and inducing a proatherogenic phenotype [86]. Several TNF-α inhibitors, such as monoclonal antibodies (infliximab, adalimumab, golimumab and certolizumab pegol) and etanercept, a dimeric fusion protein produced by recombinant DNA technology, have been developed and introduced against inflammatory and autoimmune disorders. Studies have shown that anti-TNF-α medicines improve endothelial function, aortic stiffness and vascular wall properties and decrease the risk of cardiovascular events in rheumatological patients or those with psoriasis. However, in patients with severe cardiovascular disease, TNF-α inhibitors failed to show clinical benefits. To the best of our knowledge, currently there are no ongoing clinical trials focused on the use of anti-TNF-α agents in patients with high cardiovascular risk [87,88].

### 4.2. Anti-P-Selectin Therapy

P-selectin is another interesting therapeutic target in atherosclerosis and vascular diseases. It is an inflammatory adhesion molecule strongly expressed on the surface of activated platelets and endothelial cells. P-selectin facilitates recruitment and attachment of circulating leukocytes to vascular walls and promotes proinflammatory cytokine release and thrombus formation [89]. Therapeutics targeting P-selectin are being intensively investigated in preclinical and clinical studies. Among them, inclacumab, a fully human IgG4 monoclonal antibody, is a very promising agent. It inhibits P-selectin activity and showed anti-cell adhesion, anti-inflammatory and antithrombotic effects in patients with cardiovascular diseases. The SELECT-ACS trial found that inclacumab significantly reduced myocardial damage after PCI in NSTEMI patients [12]. The drug appears to be well-tolerated, and new clinical trials with inclacumab are planned [90].

### 4.3. Angiopoietin Like (ANGPTL3) Targeting Agents

ANGPTL3 is a secretory protein belonging to the angiopoietin-like protein family. Due to its crucial role in human lipoprotein metabolism by inhibition of lipoprotein and endothelial lipases, ANGPTL3 has emerged as a promising target in cardiovascular and metabolic therapy [91]. Inhibition of ANGPTL3 activity leads to an important reduction in all major lipoprotein types, and loss-of-function (LOF) variants of ANGPTL3 were associated with a protective role against cardiovascular disorders, decreasing the risk of coronary heart disease by 34% [92]. Evinacumab is a recombinant human monoclonal antibody that inactivates circulating ANGPTL3 via the formation of complexes with the protein. It reduces plasma TG, LDLc and very-low-density lipoprotein cholesterol (VLDLc) levels, augmenting the clearance of TG-rich lipoproteins. In 2021, evinacumab was approved by the FDA for treatment of homozygous familial hypercholesterolemia [12,93]. An intensive therapy based on evinacumab, alirocumab and atorvastatin, targeting all apoB-containing lipoproteins, may provide an improved anti-atherosclerotic approach. This combination inhibited the progression of atherosclerotic lesions, reducing the area, size and proliferation of macrophages in plaque [94].

Alongside evinacumab, a similar reduction in atherogenic lipoproteins was obtained in human subjects with vupanorsen (AKCEA/IONIS-ANGPTL3-L_RX_), a N-acetyl galactosamine-conjugated second generation antisense oligonucleotide. It targets hepatic ANGPTL3mRNA interacting with the asialoglycoprotein receptor and can develop favorable cardiometabolic effects in patients with atherosclerosis [95]. Although the product met the primary endpoint of the Phase IIb TRANSLATE-TIMI 70 clinical trial (that included statin-treated subjects with dyslipidemia), some current programs of clinical development for vupanorsen have been discontinued due to safety issues related to hepatic side effects [96].

### 4.4. Photodynamic Therapy (PDT) 

PDT is an emerging minimally invasive procedure used to treat various oncologic, infectious and non-oncologic diseases. In recent years, PDT has attracted attention as an interesting therapeutic option in atherosclerosis. It induces the stabilization and regression of atherosclerotic plaques and promotes vascular healing. Macrophage depletion, decrease in foam cell content and repopulation of plaques with non-proliferating smooth muscle cells contribute to the plaque stabilizing effects induced by PDT. Besides, PDT can prevent restenosis following clinical coronary angioplasty. The procedure involves three components: photosensitizer, light with a specific wavelength and molecular oxygen. The photosensitizer specifically accumulates in the atherosclerotic plaques and, after light activation, triggers a photochemical response that includes the generation of ROS and interference of cell survival and remodeling processes [97,98,99,100]. A number of conventional photosensitizers, namely porphyrins (hematoporphyrin derivative, Verteporfin), phtalocyanine derivatives, chlorins (Talaporfin sodium), 5-aminolevulinic acid and motexafin lutetium showed phototherapeutic properties in atherosclerosis in preclinical studies [101,102,103,104,105,106,107,108,109,110]. However, translation of PDT to clinical use warrants further studies with experimental models similar to human coronary atherosclerosis, clarification of some issues related to the appropriate type, toxicity and dosage of photosensitizer, light hypersensitivity and cutaneous photosensitivity, interference with arterial calcification and lipid-lowering/antiplatelet drugs and selective delivery in the atherosclerotic area [97,98,99]. Novel nanotechnology-based delivery systems and intravascular administration (balloon catheters) of photosensitizers allow targeted, selective therapy and minimization of side effects. Polymer nanoparticles, self-assembled protein nanostructures and liposome-based formulations were suggested as possible delivery strategies. Further, photosensitizer targeting could be increased by conjugation with various ligands for class A scavenger receptors or dextran receptors localized on macrophage surfaces. Significant improvements in laser technology may also support the optimization of PDT and its translation to clinical settings [97,99].

### 4.5. Theranostics

Theranostics constitute an innovative strategy integrating diagnosis and therapy in a single delivery agent. Although the application of theranostics in cardiovascular diseases is still in its infancy, the field has attracted increasing attention, and research on the development of these agents is growing. Theranostic medicine allows targeted therapy, enhances drug effectiveness and provides efficient, image-guided and personalized treatment of diseases [111,112]. 

Advanced imaging techniques (magnetic resonance imaging (MRI), computed tomography (CT), photoacoustic imaging, positron emission tomography (PET), optical coherence tomography (OCT) and laser device imaging (LDI)) and various nanocarriers (inorganic particles (metal oxides, gold, silver and silica), lipid-based nanoparticles, polymeric nanoparticles, dendrimers or carbon nanotubes) have been studied in theranostic therapy of atherosclerosis and vascular diseases [113,114,115]. Macrophages are the most-used target for theranostics, and nanomaterials are conjugated with binding ligands such as peptides or antibodies for this purpose [115].

Iron oxide nanoparticles, paramagnetic perfluorocarbon nanoparticles conjugated with fumagillin, cerium oxide and iron oxide nanocomposites, solid lipid nanoparticles and HDL-like magnetic nanostructures were designed mainly as MRI contrast agents [116,117,118,119,120,121,122]. Among them, iron oxide nanoparticles appear to be the most promising materials for MRI treatment. Their conjugation with antibodies targeting macrophages proved their ability to visualize atherosclerotic plaques [115,123,124]. An intrinsic affinity of the atherosclerotic plaque for macrophages and the lack of immunoreactions were noticed for HDL-like nanoparticles. They stimulated cholesterol efflux and regression of the atherosclerotic plaque. Theranostic systems comprised of MRI, solid lipid nanoparticles and ultrasmall superparamagnetic iron oxide particles loaded with prostacyclin inhibited platelet aggregation, and iron nanocomposites quenched ROS in inflammatory macrophages [122].

Gold nanoparticles were mostly used as CT contrast agents, damaging inflammatory macrophages. Further, copper sulfide nanoparticles conjugated with monoclonal antibody targeting transient receptor potential cation channel subfamily V member (TRPV1) provided feasible agents for photoacoustic imaging in atherosclerosis. They are able to detect and reduce lipid storage and atherosclerotic lesions in vivo [125]. For improved detection and visualization of atherosclerotic plaques, hybrid nanosystems combining imaging techniques (PET-MRI, PET-CT) and different associated nanoparticles with multimodal properties (such as magneto-fluorescent nanoparticles with high affinity for endothelial cells, dextran-coated magnetofluorescent iron oxide nanoparticles labeled with PET tracer ^64^Cu and HDL-mimicking nanoparticles) have been suggested [125,126,127]. 

Although theranostics provides rapid and noninvasive diagnosis of atherosclerosis in animal models, development of clinically acceptable theranostics implies further research to solve some drawbacks related to design, systemic toxicity and stability of nanomaterials, the use of experimental models closer to human atherosclerosis and industrial production [115].

## 5. Natural Products with Anti-Atherosclerotic Properties 

Medicinal plants and their bioactive compounds are considered as alternative preventive and therapeutic strategies in atherosclerosis as well as a valuable resource for pharmaco-research. Many plant products found in the daily human diet promote normal cardiovascular physiology and reduce the impact of conventional cardiovascular risk factors. There is also strong preclinical and clinical evidence that some plant products are capable of exerting significant anti-atherosclerotic effects. They can attenuate oxidative stress, protect against lipid peroxidation, positively modulate HDLc function, develop lipid-lowering effects, suppress proinflammatory signaling pathways and mediators, improve endothelial function, inhibit foam cell formation and reduce the severity and progression of atherosclerotic lesions (Table 1) [128]. In addition, plant products can modulate human gut microbiota to a healthier phenotype by revitalizing beneficial phylotypes (*Bacteroides*, *Lactobacillus*, *Bifidobacterium* and *Akkermansia*) and reducing proatherogenic commensals (*Clostridium*, *Prevotella* and *Desulfovibrio*) [129]. The mechanisms of action are multiple and versatile, and the same product can exert pleiotropic effects. The most-promising medicinal plants and phytocompounds with anti-atherosclerotic properties are presented in Table 1. 

Among plant bioactives, phenolic compounds (flavonoids, stilbenes, anthocyanins and phenolic acids) appear to more potently modulate simultaneous signaling and mechanistic pathways in atherosclerosis. Further, dietary intervention trials support their protective effect in cardiovascular risk [148]. Daily intake of more than 29 mg of flavonoids may lead to a 68% reduction in the occurrence of major cardiovascular events (after correction of some known cardiovascular risk factors, such as smoking, sedentary lifestyle, obesity and high blood pressure) [149]. Garlic and Chinese sage preparations have been intensively studied and showed the most obvious atheroprotective properties. Despite strong preclinical evidence, clinical trials reported heterogeneous or inconsistent results for some plant products. This can be explained by great interindividual variability, methodological shortcomings, product diversity in terms of botanical origins, chemical characterization and lack of standardization, dosage, study period and short follow-up. Genetic polymorphisms and functionality of gut microbiota also contribute to variability of clinical outcomes. Controlled and multicentric trials of plant-derived products with large sample size and robust criteria for selection of subjects, as well as long-term evaluation, are needed to substantiate their potential clinical use in atherosclerosis.

## 6. Conclusions and Future Perspectives

Our review provides updated information on atherosclerosis and current pharmacotherapeutic approaches. In spite of remarkable progress achieved in understanding the mechanisms underlying the complex pathogenesis of atherosclerosis, the development of successful anti-atherosclerotic therapies remains a challenging task. Current therapeutic interventions are focused on improving lipoprotein metabolism and on modulation of atherosclerosis progression. Apart from statins, emerging therapies based on some cytokine-targeted agents (mainly, monoclonal antibodies) are also included in clinical practice. Another interesting emerging strategy is theranostics, which allows simultaneous treatment and imaging, promising an efficient approach to atherosclerosis and cardiovascular diseases. However, theranostics needs additional research, especially encompassing systemic toxic effects and the nanomaterial manufacturing process prior to clinical use. The establishment of chemopreventive strategies in atherosclerosis is also of paramount importance since prophylaxis is more beneficial than trying to reduce atherosclerotic lesions and plaque. The inclusion of documented and well-characterized plant products as components of these preventive programs could be a valuable intervention. Besides the effect of standardized plant products as an add-on therapy to conventional medicines, they could be an interesting avenue for the development of new therapeutics. The design of personalized treatments based on the concept of so-called “network medicine”—that includes the analysis and integration of genetic, metabolic and epigenetic factors—has been suggested as a more effective and appropriate therapeutic strategy in atherosclerosis [12].

## Figures and Tables

**Table 1:** Natural products and their anti-atherosclerotic effects.

Plant Products	Bioactives/Chemical Class	Anti-Atherosclerotic Effects	Putative Mechanisms	Clinical Studies	References
*Allium sativum*, garlic	Organo-sulfur derivatives	Lipid-lowering LDLc oxidation inhibition VSMC antiproliferative CIMT inhibition Inhibition of cholesterol accumulation in arterial wall Endothelial protective Anti-inflammatory Antithrombotic Antihypertensive	↓ACC, ACAT, HMGR, (SREBP)-1c, G6PD ↓TNF-α, IL-1β, COX-2 ↓CAM-1, HLA-DR ↓TXB2, PGE2, leukotriene C4 ↓GPIIb/IIIa receptor, fibrinogen binding ↓sialidase Regulation of NO synthesis ↓AngII receptor	Heterogeneous results on blood lipid profile Reduction in LDLc, TC in patients with hypercholesterolemia if it is used for longer than 2 months Reduction (by 38%) in risk of coronary events Decrease of blood pressure in hypertensive subjects Decrease of CIMT and regression of plaque in patients with carotid atherosclerosis, coronary heart disease	[128,130,131]
Berberine	Isoquinoline alkaloid	Endothelial protective Lipid regulator Plaque-stabilizing Decrease of foam cell formation Increase of macrophage autophagy Anti-inflammatory Gut microbiota modulation	↑LDL receptors, apoE expression ↓HMGR ↓NF-κB, TNF-α, MCP-1, IL-6, MMP-9 ↓p38-MPK ↑LXR-α, ABCA1 ↑AMPK-SIRT1-PPARγ ↑AMPK/mTOR ↓PI3/Akt/mTOR	Reduction in serum cholesterol in patients with hypercholesterolemia Decrease of atherosclerotic area	[129,132,133]
Curcumin	Diarylheptanoids	Anti-inflammatory Endothelial protective Lipid-lowering and regulation of lipid metabolism Plaque stabilizing Reduction of foam cell formation Decrease of macrophage infiltration Modulation of macrophage polarization Decrease of atherosclerotic lesions Decrease of carotid artery neointima formation VSMC antiproliferative Antioxidant Antiplatelet	↑cholesterol efflux ↑LXR-ABCA1/SR-BI ↑CD36 ↓TLR4 expression ↓oxLDL ↓NF-κB, TNF-α, IL-1β, IL-6, MCP-1, MMP-9, -13 ↓ICAM-1, VCAM-1 ↓Ang II ↑PPARγ ↑Nrf2 ↓PAF	Reduction of LDLc, TC in patients with acute coronary syndrome Decrease of hCRP, TG, TC and LDLc in patients with type 2 diabetes	[134,135,136,137]
Green tea catechins	Polyphenols	Antioxidant Endothelial protective Anti-inflammatory Lipid lowering Inhibition of oxLDL VSMC antiproliferative Decrease of plaque formation	↑Nrf2/HO-1 ↓NF-κB, TNF-α, IL-6 ↓CRP ↓TLR4 ↑IL-10 ↑AMPK/PPARγ ↑PPARα ↑markers of autophagy Regulation of LXRα, FAS, SIRT-1, Insig-1-SREBP-SCAP ↓Notch receptor ↓AngII receptor 1	Improvement of blood lipid profile (↓TC, ↓LDLc, ↓TG) in patients with mild hypercholesterolemia Improvement of endothelial function in prehypertensive subjects	[138]
*Morus alba*, mulberry	Phenolic acids Flavonoids Iminosugar alkaloids	Antihyperlipidemic LDL oxidation inhibition Decrease of lipid accumulation in foam cells Decrease of plaque volume Anti-inflammatory Antioxidant VSMC antiproliferative Antiplatelet	Regulation of FAS, GPAT, SREBP-1c, LXR ↑AMPK, PPARα AP-1, STAT3 signaling modulation ↓NF-κB, TNF-α, IL-1β, IL-6, COX-2 ↑SOD, GPx, glutathione-S-transferase ↓lipid peroxidation ↓sVCAM-1 ↓integrin α_IIb_β_3_ secretion ↓TXA2	Improvement of serum lipid profile (↓TC, ↓LDLc, ↓TG, ↑HDLc) in patients with early stage of dyslipidemia, type 2 diabetes and dyslipidemia, heart disease Reduction of atherosclerotic lesions and CIMT in patients with coronary heart disease ↓CRP levels in mild dyslipidemia	[129,139,140]
*Panax ginseng*	Triterpenes (ginsenosides)	Enhancement of plaque stability Decrease of foam cell formation Reduction of plaque formation Increase of macrophage autophagy Decrease of monocyte adhesion events Decrease of vascular calcification Attenuation of neointimal hyperplasia VSMC antiproliferative Antioxidant	↑AMPK/mTOR ↑GPER, p-PI3K ↓p38/JNK-MAPK ↑IL-4, STAT6 ↓oxLDL uptake ↑LXRα, ABCGA1 ↓NF-κB, TNF-α, MMP-2,-9 ↓VCAM-1, ICAM-1, E-selectin ↑Nrf2/HO-1, SOD	Attenuation of endothelial dysfunction in hypertensive patients Improvement of lipid profile in type 2 diabetic patients Inconsistent results in most of the studies	[129,140,141,142,143]
*Punica granatum*, pomegranate	Tannins, Anthocyanins, Flavonoids	Antioxidant Anti-inflammatory Lipid-lowering Modulation of gut microbiota	↑LXRα, ABCA1 ↓NF-κB, TNF-α ↑IL-10	Blood lipid-lowering effects in hyperlipidemic, overweight and obese subjects but also discordant results in other studies Decrease (up to 30%) of CIMT in patients with carotid artery stenosis Reduction of blood pressure in hypertensive patients with mild/high cardiovascular risk	[129,144,145]
Resveratrol	Stilbene	Antioxidant Anti-inflammatory Lipid regulator Decrease of LDLc oxidation Decrease of foam cell formation Reduction of plaque formation Vasoprotective Antiproliferative/antimineralizing	↓NADPH oxidase ↓NF-κB, TNF-α, MCP-1, IL-6, IL-8 ↓ICAM-1, VCAM-1 ↓oxLDL uptake ↑LDL receptors expression ↑CYP7A1 expression ↓HMGR ↓LOX1 ↑AMPK-PPAR ↑PPARγ ↑LXRα, ABCA1	Reduction in plasma TG in obese patients/smokers Reduction of LDLc in type 2 diabetes patients Reduction (20%) in oxLDL in patients with statins and high cardiovascular risk	[146]
*Salvia miltiorrhiza*, Chinese sage, Danshen	Tanshinones Salvianolic acids	Antioxidant Endothelial protective Anti-inflammatory Lipid regulator Reduction of foam cell formation Inhibition of progression of atherosclerotic plaque Antithrombotic Antihypertensive	↑SOD ↑NO ↓NF-κB, TNF-α, IL-1β, IL-6, MCP-1 ↓ICAM-1, VCAM-1, (MMP)-2,-3,-9 ↓oxLDL ↓CD36, PPARγ ↑ERK/Nrf2/HO-1 ↓CCl-20 ↓p38MAPKK ↑Prdx1/ABCA1 signaling ↓PI3K signaling	Improvement of blood lipid profile in patients with hyperlipidemia Reduction of blood pressure and pulse rate in hypertensive subjects Recovery of cardiac function in patients with myocardial infarction undergoing PCI	[129,147]

ABCA1, ATP-binding cassette transporter A1; ACAT, acyl-CoA cholesterol acyltransferase; ACC, acetyl-CoA carboxylase; Akt, protein kinase B; AMPK, adenosine monophosphate protein kinase; AngII, angiotensin II; AP-1, activator protein 1; Apo E, apolipoprotein E; CD36, cluster of differentiation 36, platelet membrane glycoprotein IV, scavenger receptor class B, member 3; CIMT, carotid intima-media thickness; COX, cyclooxygenase; ERK, extracellular signal-regulated kinase; FAS, fatty acid synthase; CYP7A1, cytochrome P450 family 7 subfamily A member 1; GAPT, glycerol-3-phosphate acyltransferase; G6PD, glucose-6-phosphate dehydrogenase; GPER, G protein-coupled estrogen receptor 1; GPx, glutathione peroxidase; GPIIb/IIIa, glycoprotein IIb/IIIa; hCRP, human C-reactive protein; HDLc, high-density lipoprotein cholesterol; HLA-DR, human leucocyte antigen-DR isotype; HMGR, 3-hydroxy-3-methyl-glutaryl-coenzyme A reductase; HO-1, heme oxygenase 1; ICAM-1, intercellular adhesion molecule 1; IL, interleukin; Insig-1, insulin-induced gene 1; JNK, c-Jun N-terminal kinase; LDLc, low-density lipoprotein cholesterol; LOX-1, receptor of oxLDL; LXRα, liver X receptor α; mTOR, mammalian target of rapamycin; MCP-1, monocyte chemoattractant protein-1; MMP (-2, -3, -9, -13), matrix metalloproteinase; NADPH oxidase, nicotinamide adenine dinucleotide phosphate oxidase; NF-κB, nuclear factor-κB; NO, nitric oxide; Nrf2, nuclear factor erythroid 2-related factor 2; oxLDL, oxidized LDL; p38/MAPK, p38 mitogen-activated protein kinase; PAF-2, platelet-activating factor acetylhydrolase homolog 2; PCI, percutaneous coronary intervention; PGE2, prostaglandin E2; PI3K, phosphoinositide 3-kinase; PPAR (-α, -γ), peroxisome proliferator-activated receptor; Prdx1, peroxiredoxin 1; SCAP, SREBP cleavage-activating protein; SR-BI, scavenger receptor class B type I; SIRT1, sirtuin 1; SOD, superoxide dismutase; SREBP-1c, sterol regulatory element-binding protein-1c; STAT (-3, -6) signal transducer and activator of transcription (-3, -6); TC, total cholesterol; TG, triglycerides; TLR4, Toll-like receptor 4; TNF-α, tumor necrosis factor α; TXA2, TXB2, thromboxane A2, thromboxane B2; VCAM-1, vascular cell adhesion molecule 1; VSMC, vascular smooth muscle cells; ↑, upregulation/increase of levels; ↓, downregulation/decrease of levels.

## Data Availability

Not applicable.
